# Dynamics of Attentional Bias to Threat in Anxious Adults: Bias towards *and/or* Away?

**DOI:** 10.1371/journal.pone.0104025

**Published:** 2014-08-05

**Authors:** Ariel Zvielli, Amit Bernstein, Ernst H. W. Koster

**Affiliations:** 1 Department of Psychology, University of Haifa, Haifa, Israel; 2 Department of Psychology, University of Ghent, Ghent, Belgium; University of Vienna, Austria

## Abstract

The aim of the present study was to question untested assumptions about the nature of the expression of Attentional Bias (AB) towards and away from threat stimuli. We tested the idea that high trait anxious individuals (*N* = 106; *M*(*SD*)_age_ = 23.9(3.2) years; 68% women) show a stable AB towards multiple categories of threatening information using the emotional visual dot probe task. AB with respect to five categories of threat stimuli (i.e., angry faces, attacking dogs, attacking snakes, pointed weapons, violent scenes) was evaluated. In contrast with current theories, we found that 34% of participants expressed AB towards threat stimuli, 20.8% AB away from threat stimuli, and 34% AB towards some categories of threat stimuli *and* away from others. The multiple observed expressions of AB were not an artifact of a specific criterion AB score cut-off; not specific to certain categories of threat stimuli; not an artifact of differences in within-subject variability in reaction time; nor accounted for by individual differences in anxiety-related variables. Findings are conceptualized as reflecting the understudied dynamics of AB expression, with implications for AB measurement and quantification, etiology, relations, and intervention research.

## Introduction

Selective attention to threat reflects an adaptive neurocognitive function to protect us from danger [1]–[3]. Dysregulation in this adaptive process, termed threat-related attentional bias (AB), has been linked to a maladaptive bio-psycho-behavioral cascade of information processing underlying the aetiology and maintenance of anxiety related disorders [2], [4]–[9]. Central to the present study, research has revealed two key expressions of AB – bias *towards* and bias *away* from threat cues [4], [10], [11]. Theoretical and empirical work has conceptualized these expressions of AB – towards and away from threat stimuli – as a function of (dys)regulation in the neurocognitive processes underlying attentional allocation. Specifically, it is thought that AB towards and away from threat stimuli occurs as a function of time of threat presentation where early preferential allocation of attention to threat initially is followed by more elaborated stages of information processing bias *towards* threat (due to difficulty to disengage attention) which can subsequently be followed by strategic avoidance of threat [2], [4], [11], [12]. Accordingly, this “vigilance-avoidance” pattern of attention has been used to explain why anxious individuals fail to habituate to threat despite enhanced processing of threat at early stages of information processing [13].

It has been theorized that high trait anxious individuals demonstrate a stable tendency to express AB towards threatening stimuli at the early stages of information-processing [14], [15]. In theories of anxiety-related AB it is proposed that trait anxiety is associated with a tendency to appraise novel or mildly threatening stimuli as being highly threatening, resulting in interference of ongoing behavior and AB [13], [16]–[18]. However, key aspects of such theories on threat-related AB in anxiety are untested, yet may be important to understanding the nature of AB as well as its measurement and study. Most notably, if theory regarding the mechanisms of threat-related AB is correct, then trait anxious individuals should allocate attention to a *broad range* of threatening information. However, the degree to which AB is indeed a stable phenomenon that is expressed similarly with respect to different classes of threatening stimuli has received limited empirical study. Relatedly, extant study of AB has presumed that a person may exhibit either AB towards *or* AB away from threatening stimuli, in a mutually exclusive manner [2], [4], [11], [12]. Yet, direct empirical evidence for this cardinal assumption is lacking.

In the present study, we propose that the expression of AB to threat could be more complex than has been recognized by current theories [13], [16]. We posit that a person who demonstrates AB towards one category of threat has the potential to exhibit an alternative attentional response to other categories of threatening stimuli (e.g., angry faces). This proposal is based on understanding of the mechanisms underlying AB towards and away from threat, and related work on other forms of self/emotion-regulation. Specifically, we theorize that variability in attentional responding to different classes of threatening stimuli may be observed due to a number of factors: (1) differential emotional responding to different categories or forms of threatening stimuli [19]–[21], (2) differential motivational relevance of different threatening stimuli [22], [23], (3) past learning specific to certain forms of threat [23]–[25], (4) and/or more broadly, yet unknown contextual factors that may moderate the self/emotion-regulatory attentional mechanisms by which a person may respond to different threatening stimuli [26].

We therefore sought to examine different possible patterns of AB in high trait anxious individuals where individuals could show: (a) No AB, (b) AB towards one or multiple categories of threat stimuli, (c) AB away from one or multiple categories of threat, and (d) AB towards one or multiple threat categories (e.g., angry faces, violent scenes) and AB away from one or multiple other threat categories (e.g., attacking dogs, attacking snakes). To simplify subsequent communication regarding this prediction in the manuscript, we termed the prediction that different individuals could express different patterns of AB the *Multiple Expressions of Attentional Bias* Hypothesis (MEAB).

Several methodological choices that guided the present study are noteworthy. First, trait anxiety, as a function of the Spielberger Trait-state Anxiety Inventory (STAI) [27] scores, was selected as a marker of AB to threat for a number of reasons. Theories of AB to threat propose that individual differences in trait anxiety are linked to AB towards threat [13], [16]; the majority of published studies examining anxiety and AB to threat have focused on trait anxious adults using the STAI [4] – facilitating comparison to past work. Second, we examined the MEAB using one of the most commonly used behavioral tasks to assess AB, the dot probe task [28]. In this task, two stimuli (often one threatening and one neutral) are presented on different sides of a screen where one of the stimuli is followed by a probe at its location. Selective attention towards a motivationally-relevant stimulus will speed reaction time (RT) when a probe appears in the spatial location of the target stimulus and slow RT when a probe appears in the spatial location opposite of the target stimulus (i.e, AB *towards*); and vice-versa when selective attention is allocated away from the motivationally-relevant stimulus (i.e., AB *away*). Third, various stimulus durations of threat have been used in this task in past research (e.g., 250 ms, 500 ms, 750 ms, 1500 ms). To rigorously test the MEAB hypothesis, we measured AB using a 500 ms stimulus duration that has been: (a) most extensively studied [4], [9], [29]; (b) sufficient in duration to permit looking towards followed by looking away [30], [31]; (c) characterized predominantly by AB towards threat in extant work [31]; and (d) brief enough to provide a maximally rigorous test – a longer duration would increase the likelihood of observing both towards and away [32], and a shorter duration may reduce the likelihood of observing AB away [32], [33].

## Method

### Participants

One-hundred-six trait anxious (State- Trait- Anxiety Inventory (STAI)-Trait Anxiety scores >42; [34]–[36]) young adult participants (M(SD)_age_ = 23.9(3.2) years-old, range_age_ 18–37; 67.9% female) were recruited from a university community in Israel. The identified STAI cut-off reflecting elevated trait anxiety is based on a local (Israeli) norm reflecting 1*SD*<STAI mean among a treatment-seeking (mood/anxiety disorders) clinical population (N = 275) and 1*SD*>STAI mean among healthy control participants (N = 534) [37]. In addition, potential participants were excluded on the basis of the following criteria: (a) impaired eyesight (uncorrected); (b) lack Hebrew-language reading and speaking fluency; or (c) current psychopharmacological treatment for anxiety or depression due to possibility that these agents may threaten the internal validity of the study by impacting estimates of attention bias. The study through which the proposed data was collected received human subjects research ethics approval through a University of Haifa Department IRB committee. Participants provided their written informed consent to participate in this study. The IRB approved this consent procedure.

### Measures

Measures were translated from English to Hebrew and then back-translated by a separate party using structured guidelines [38], [39]. We administered the *STAI* [35], *Overall Anxiety Severity and Impairment Scale* [40], *Inventory of Depression and Anxiety Symptoms* (IDAS) [41], and the *Anxiety Sensitivity Index-3* (*ASI-3*) [42].

#### Attentional Bias Measurement

The visual emotional dot probe task [28], [43] was used to measure AB. Participants were presented with a fixation cross (500 ms), followed by 250 ms blank screen, followed by two stimuli presented simultaneously for a duration of 500 ms. One stimulus was presented to the left of the fixation cross and the other to the right, one of which was immediately replaced by a small black probe that was randomly presented to the right or left. Participants were instructed to first focus their gaze on the fixation cross and then, as quickly and accurately as possible, press one of two (left or right) response box buttons using their left- and right- index fingers corresponding to the location of the probe. A random interval of 500–1500 ms preceded the next trial. On incongruent trials (IT), the probe appeared in the location of the neutral stimulus, whereas on congruent trials (CT) the probe appeared in the location of the threat stimulus. Additional trials include neutral-neutral stimuli presentation (No Threat: NT). The task included 160 trials, randomly distributed to 40 CT, 40 IT and 80 NT. Sixteen trials (IT and CT) represented each of 5 categories of threat stimuli, also randomly distributed across task trials.

### Procedure

Potential participants completed the trait anxiety section of the STAI. The identified sub-sample of high-anxious participants completed an online battery of self-report measures (see above) and then attended a single laboratory session. Participants were told that task instructions would be delivered via instructions on the computer monitor throughout the experiment. Participants then completed the emotional visual dot probe task to measure levels of AB to threat. This task consisted of 15 practice trials and 160 test trials. Participants were debriefed and compensated via course credit or a small payment.

### Materials and Apparatus

#### Stimuli

Based on extant AB and emotion elicitation literatures [4], [19], we operationally defined threat stimuli as images perceived as threatening and which elicit acute anxiety or fear in participants. Five categories of threat stimuli were sampled from the AB and emotion elicitation literatures (i.e., angry faces vs. attacking dogs vs. attacking snakes vs. pointed weapons vs. violent scenes). Each category reflects a distinct semantic category of threatening stimuli as well as a variety of characteristics that may further distinguish between various forms of threat (i.e., natural or artificial, social or nonsocial, human or non-human, evolved fear or learned fear stimuli). Threat and neutral stimuli were, when available, selected from the International Affective Pictures System [44], guided by published studies of threat-related bias and fear elicitation, and digitally resized to 8-cm width×5-cm width-height [17], [45]–[47]. We also selected additional images online when the IAPS did not include a sufficient number of stimuli for each of the threat categories (18 IAPS images, 12 images selected online; stimulus materials are available upon request to the corresponding author). Neutral stimuli were matched to threat stimuli with respect to image content (e.g., hand holding pen matched with hand holding gun), complexity, luminance, and contrast. Selection of stimuli was further guided by pilot testing to ensure that stimuli were experienced as threatening and elicited fear, specifically. Selected stimuli were rated by an additional sample of N = 20 independent raters (university students), who were asked to rate how threatening the stimuli were (1 = *not threatening at all* to 5 = *extremely threatening*) and to what degree (1 = *not at all* to 5 = *very strongly*) a list of emotions were experienced upon viewing a picture (see Tables 1–2).

**Table 1:** Perceived Threat Ratings among Independent Sample (N = 20).

	Neutral	Threat*	Dogs	Faces	Snakes	Weapon	Violence
Mean (SD)	**1.07 (0.15)**	**3.01 (0.79)**	3.14 (0.96)	2.22 (0.87)	3.27 (1.05)	3.05 (1.02)	3.36 (0.81)
Observed Range	**1–1.67**	**1.73–4.27**	1.67–4.83	1–4.33	1.5–5	1.67–5	2.33–5

Note. *Threat = Mean of perceived threat ratings across all five categories of threat stimuli.

**Table 2:** Emotion Intensity Ratings for Threat Stimuli among Independent Sample (N = 20).

	Sadness	Anger	Disgust	Anxiety	Guilt	Embarrassment	Interest	Joy	Amusement	Love	Cheerfulness
Mean	1.30	1.43	1.56	**2.53**	1.03	1.02	1.26	1.02	1.06	1.00	1.01
(SD)	(0.25)	(0.42)	(0.4)	**(0.65)**	(0.1)	(0.06)	(0.23)	(0.06)	(0.11)	(0)	(0.04)
ObservedRange	1–1.8	1–2.6	1–2.2	**1.6–3.8**	1–1.4	1–1.2	1–1.6	1–1.2	1–1.4	1–1	1–1.2

#### Experimental set-up

The experiment was run via E-Prime experimental presentation software [48]. The experimental session was conducted on a Hewlett-Packard computer and 19″ CRT monitor, in an acoustically-insulated room, with a one-way observation window. Participants’ responses were measured via Psychology Software Tools Serial Response Box.

### Data Preparation

#### Bias Scores

We computed bias scores (BS) by subtracting Mean Response Time (RT) of congruent threat trials from Mean RT of incongruent threat trials [4]. This BS was computed per participant across all categories of threat cues per participant (i.e., one BS for all threat categories) as well as per category of threat per participant (i.e., one BS for each of 5 threat categories).

#### Attentional Bias Status and Direction Per Threat Category

**Figure 1:**
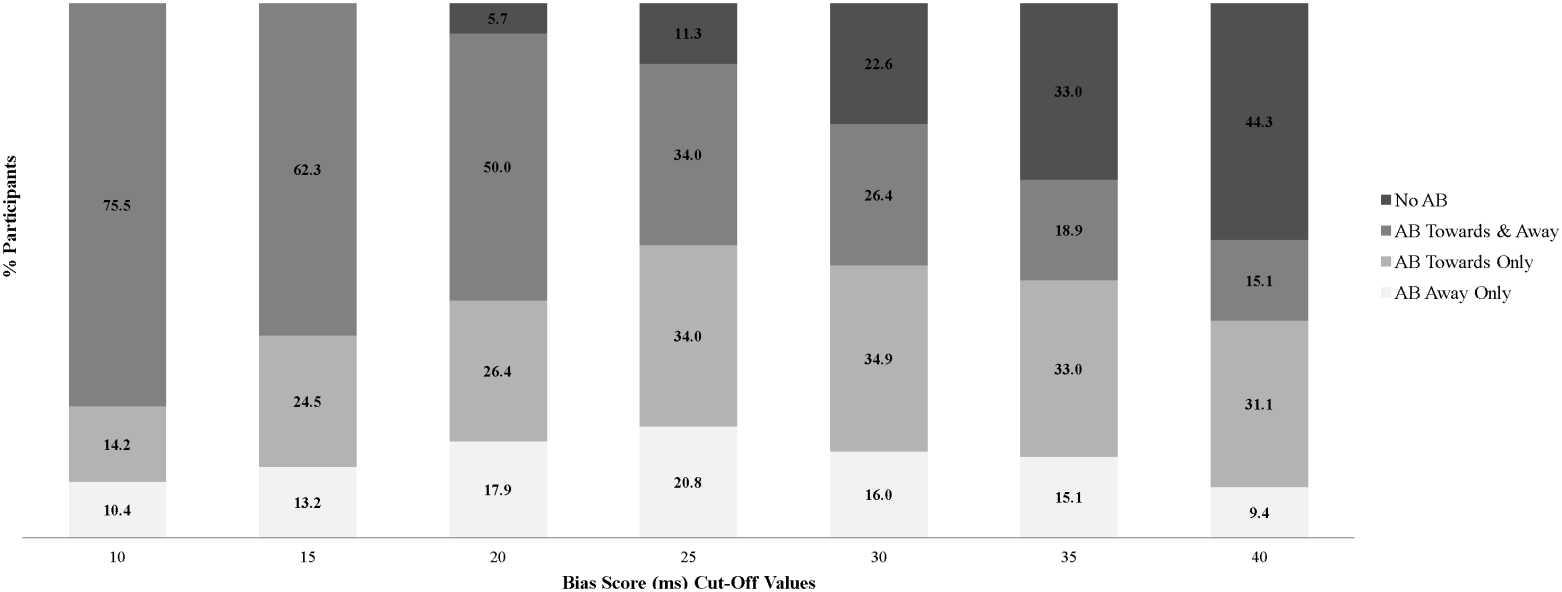
Rates of the Multiple Expressions of Attentional Bias to Threat as a function of Bias Score Cut-Off Values among Anxious Participants (N = 106).

We first classified each participant’s BS, per threat category, as reflective of either no AB, AB towards, or AB away. To do so, we operationalized a conservative criterion value to classify attentional responding to threat stimuli as evidence of AB. Specifically, we preliminarily defined BS>25 ms as the criterion for AB towards and BS<−25 ms as the criterion for AB away, per threat category (no AB was thus defined as BS>−25 ms **and** BS<25 ms). We identified this initial 25 ms criterion cut-off value for a number of reasons: (1) first and foremost, this is a conservative operational definition of AB as few AB researchers are likely to question whether BS scores greater than that criterion value likely reflect no AB; (2) AB bias scores as small as 10 ms are regularly interpreted as evidence of AB [2], [4], [9], [49], [50]; (3) we tested alternative less versus more conservative criterion values (10 ms to 40 ms) (Figure 1); based on these analyses, we selected 25 ms as it reflected empirically the AB criterion cut-off value which maximized the prevalence of towards/away-only sub-groups thereby providing the most rigorous test of the proposed MEAB hypothesis and specifically reducing the probability of observing participants expressing AB towards *and* away from different threat categories artifactually, due to the selected cut-off (see Results below for details); furthermore, as illustrated in Figure 1, the key conclusions regarding the expressions of AB do not change as a function of the specific cut-off value; (4) Finally, larger or even more conservative criterion cut-off values (e.g., 40 ms) increase the probability of false-negatives or misclassification of cases likely to in fact demonstrate robust AB to the no-AB (<40 ms) sub-group (see Figure 1).

#### Data Reduction

RT outliers (trial RT<200 or >1500 ms, trial RT> or <3 *SD*s of participant’s mean RT; *M(SD)* = 2.9 (3.2) outlier trials/participant, (<1% trials/participant) and error-response trials (i.e., “left” response when probe appeared on the right; *M(SD)*_errors_ = 0.7(1.1), <1% of trials) were discarded, based on a priori criteria for valid trial selection [4].

## Results

### I. Perceived Threat and Anxiety Ratings by Threat Category

Prior to testing AB to threat stimuli, it is important to explore differences between threat categories with respect to their perceived level of threat. Table 1 presents mean levels of perceived threat scores per studied threat categories. Overall, among an independent sample of young adult participants (N = 20), threatening faces were rated as less threatening than all other categories of threat (*t*_(19)_ >3.8, *p*<.001); violence was perceived as more threatening than weapons (*t*_(19)_ = 2.2, *p* = .04); and no other significant differences were found between weapons, attacking snakes, or attacking dogs in terms of perceived threat (*t*_(19)_ <1.3, *n.s*). Furthermore, as expected, overall the threatening stimuli were rated as significantly more threatening than emotionally-neutral stimuli (*t*_(19)_ = 11.5, *p*<.001). Additionally, threatening stimuli were rated as eliciting significantly elevated levels of anxiety relative to all other negatively-valenced emotions including sadness, anger, disgust, guilt, and embarrassment (*t*_(19)_ = 8.88, *p*<.001) and positively-valenced emotions including, interest, joy, amusement, love, and cheerfulness (*t*_(19)_ = 10.17, *p*<.001).

### II. Traditional Sample-Level Attentional Bias

First, in line with traditional computation of sample-level AB, we tested the direction, magnitude and statistical significance of AB. Sample-level mean(SD) AB = 6.5(15.7) ms towards threat, across all categories of threat stimuli, was significantly larger than 0 (i.e., no bias; *t*_(105)_ = 4.25, *p*<.001, Cohen’s *d* = .41). Sample-level bias was then similarly tested by threat category; significant bias towards each threat category, with exception of threatening dogs was observed (M_Dogs_ = 2.5(30.3), *t*_(105)_ = .85, *n.s*; M_Faces_ = 8.1(33.1), *t*_(105)_ = 2.5, *p* = .01, Cohen’s *d* = .24; M_Snakes_ = 7.1(32.0), *t*_(105)_ = 2.28, *p* = .03, Cohen’s *d* = .22; M_Weapon_ = 6.9(29.2), *t*_(105)_ = 2.4, *p* = .02, Cohen’s *d* = .24; M_Violence_ = 7.9(33.5), *t*_(105)_ = 2.42, *p*<.02, Cohen’s *d* = .24).

### III. Multiple Expressions of Attentional Bias

We then tested the MEAB hypothesis in these data. Based on the AB>25 ms and AB<–25 ms criterion cut-offs, participants expressed the following patterns of AB: a) 34% of participants demonstrated AB towards one or more threat categories (BS>25 ms), and in all other categories demonstrated BS<25 ms and BS>–25 ms (group “AB *towards* threat *only*”). b) 20.8% of participants demonstrated AB away from one or more threat categories (BS<–25 ms), and in all other categories demonstrated BS<25 ms and BS>–25 ms (“AB *away* from threat *only*”). c) 34% of participants demonstrated AB towards one or more threat categories (BS>25 ms) and concurrently AB away from one or more other threat categories (BS<–25 ms) (“AB towards *and* away from threat”). d) 11.2% of participants demonstrated neither AB towards nor away from any category of threat stimuli (BS<25 ms and >–25 vms) (“No AB”). See Table 3 for a summary of BS by observed sub-groups of AB expressions.

**Table 3:** Observed Expressions of Attentional Bias: Descriptive Statistics.

		Total	Attentional Bias Expressions			
			Towards Only	Away Only	Towards & Away	No Bias
			T>0, A = 0	T = 0, A>0	T>0, A>0	T = 0, A = 0
	% (N)	100 (106)	34 (36)	20.8 (22)	34 (36)	11.2 (12)
	% Female	67.9	55.6	68.2	77.8	75
Mean BS (SD)	Across Categories	6.5 (15.7)	19.0 (14.8)	–11.5 (5.9)	6.2 (10.9)	2.6 (6.1)
	Towards Categories*	43.7 (14.3)	43.0 (16.1)	X	44.4 (12.5)	X
	Away Categories**	–40.9 (13.7)	X	–39.7(15)	–41.6(13)	X

*Note*. BS = Incongruent-Congruent trials RT.

*Across Categories: Mean BS across all 5 categories of threat stimuli.

*Towards Categories: Mean BS in categories for which each individual demonstrated AB towards (BS>25 ms).

**Away Categories: Mean BS in categories for which each individual demonstrated AB away (BS<−25 ms).

T = #of categories with BS>+25 ms (towards categories);

A = #of categories with BS<−25 ms (away categories).

**Figure 2:**
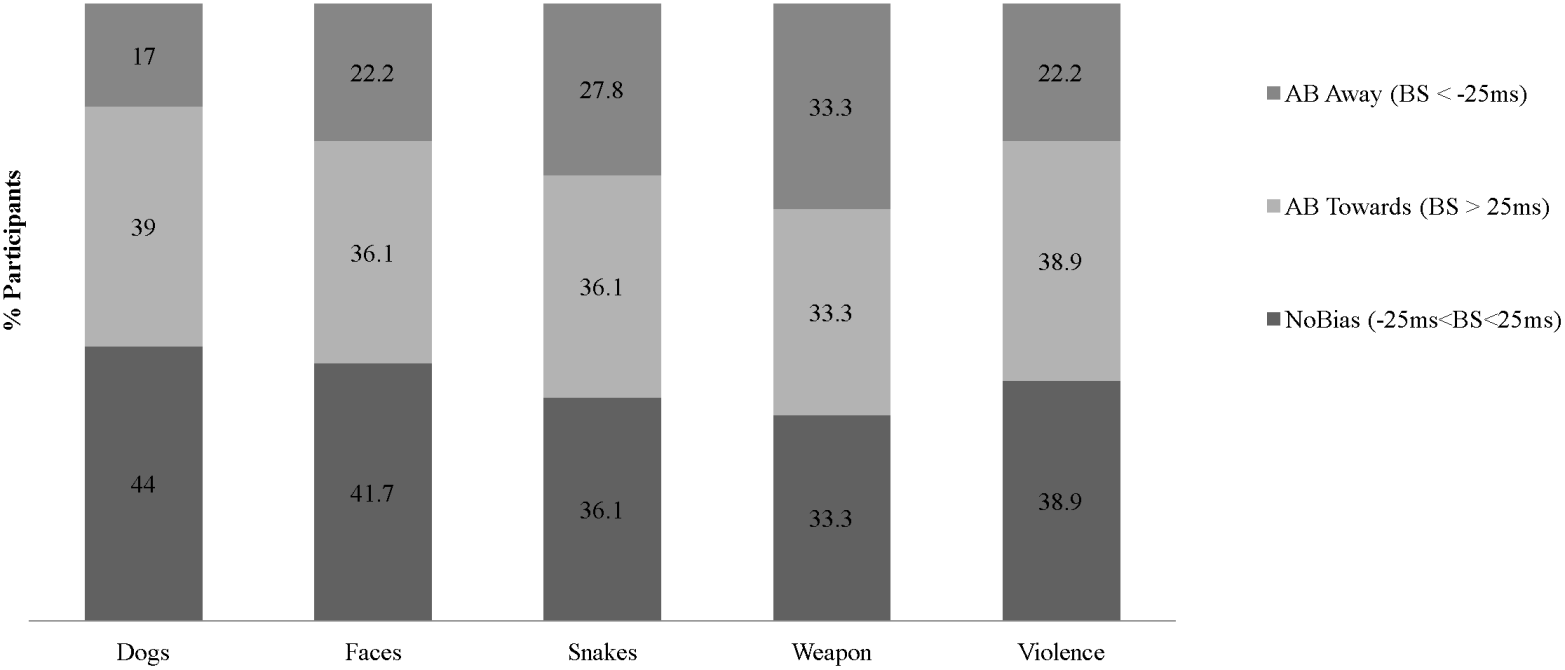
Attentional Bias by Threat Category among Participants Demonstrating AB Towards & Away from Different Categories of Threat Stimuli (N = 36).

**Figure 3:**
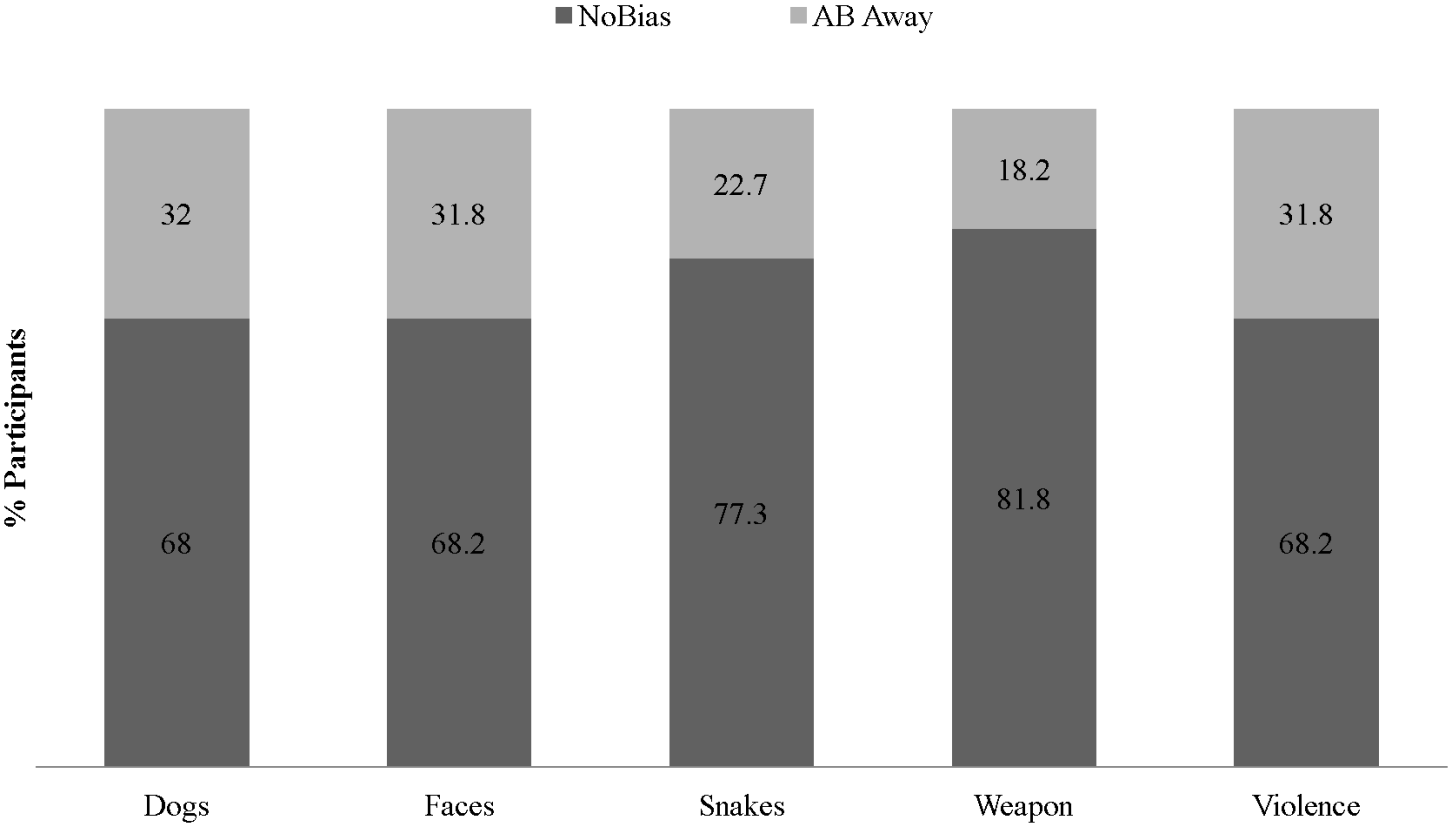
Attentional Bias by Threat Category among Participants Demonstrating AB Away-Only (N = 22).

**Figure 4:**
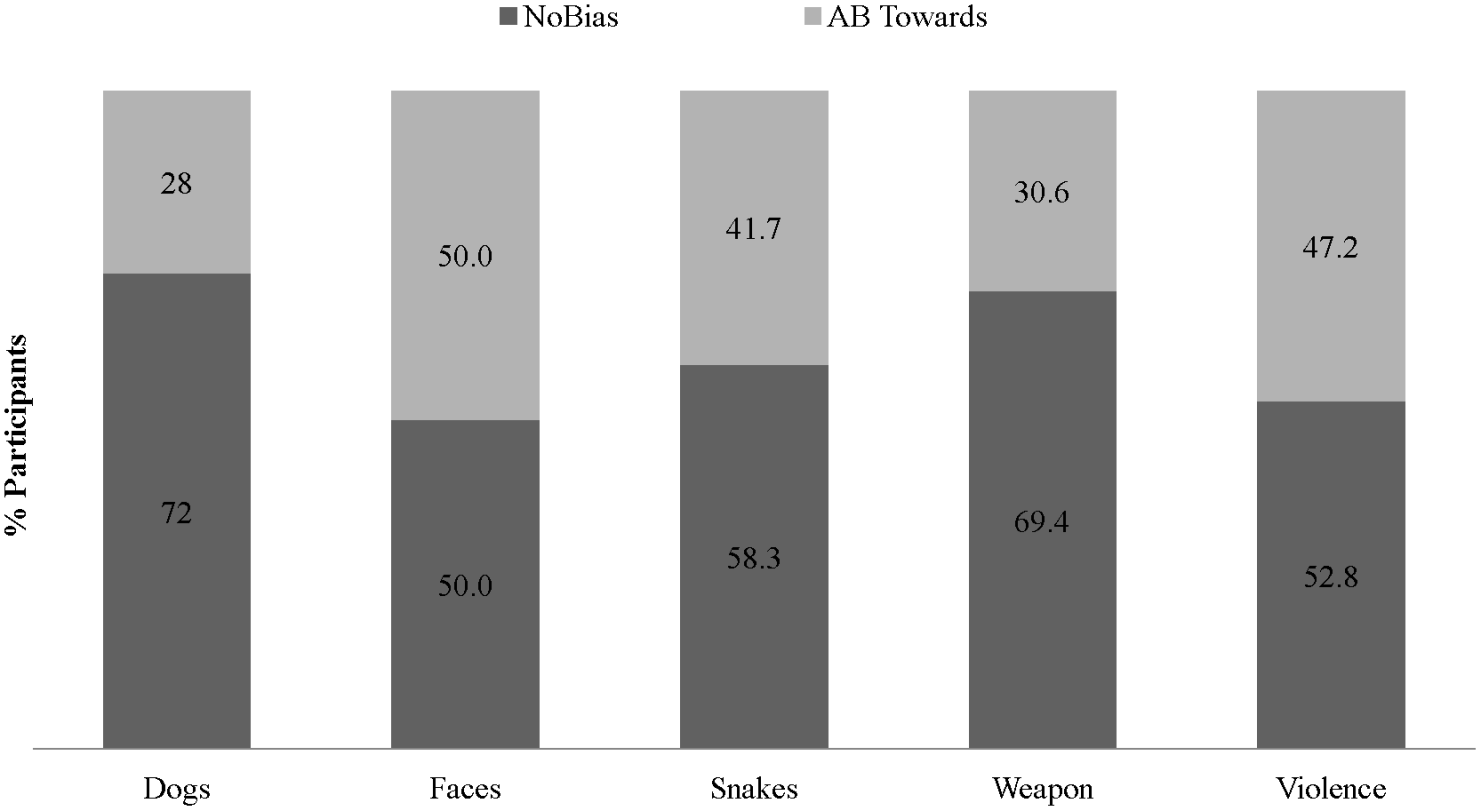
Attentional Bias by Threat Category among Participants Demonstrating AB Towards-Only (N = 36).

Three additional analyses were designed to test and thereby rule-out alternative explanations of the observed expressions of AB. First, we rule out that the observed expressions of AB are a by-product of the specific criterion cut-off selected (see Figure 1 for evidence of multiple AB expressions across a range of BS criterion cut-off values). Second, we rule out that the observed multiple expressions of AB are a by-product of the specific categories of threat selected or only observed with respect to certain but not all categories of threat. Specifically, Figures 2, 3, and 4, respectively illustrate that AB towards *and* away from different categories of threat concurrently, AB away from threat only, and AB towards threat only, are not specific to nor explained by any specific category(ies) of threat. Third, we rule out that AB towards *and* away from different categories of threat concurrently was a methodological artifact of greater within-subject variability in RT, relative to participants demonstrating AB towards threat only or AB away from threat only. No significant differences were found between the *mean SD of the RTs in categories wherein AB towards was expressed* between participants demonstrating AB towards & away (*M*(*SD*) = 115(68.2)) and those demonstrating AB towards only (*M*(*SD*) = 94.3(67.8) *t*_(57)_ = 1.2, *n.s*); nor differences between the *mean SD of the RTs in categories wherein AB away was expressed* between participants demonstrating AB towards & away (*M*(*SD*) = 78.6(32)) and those demonstrating AB away only (*M*(*SD*) = 66.8(28.6) *t*_(47)_ = 1.3, *n.s*).

### IV. Anxiety-related Variables and Attentional Bias Expressions: Follow-up Exploratory Analysis

See Table 4 for descriptive statistics for levels of anxiety-related variables by AB expressions. Overall, no differences in anxiety-related variables were observed between participants expressing each of the expressions of AB (towards only, away only, towards & away; *F*_(1,57)_ = 1.16, *n.s*). Furthermore, the number of participants demonstrating clinical levels of anxiety (OASIS total score >7; [51] did not differ between participants with mixed bias expressions (*N* = 9/36) relative to participants with AB towards only (*N* = 6/36; *χ^2^ = *0.66, *n.s*).

**Table 4:** Levels of Anxiety-Related Problems by Attentional Bias Expressions.

	Towards Only	Away Only	Towards & Away
	(n = 36)	(n = 22)	(n = 36)
	*M (SD)*	*M (SD)*	*M (SD)*
STAI - Trait Anxiety	49.5(6.4)	46.8(5.5)	47.6(5.5)
OASIS - Total	4.9(3.3)	3.4(3.3)	5(2.9)
IDAS - Panic	12.8(4.5)	10.2(3)	14.1(5.3)
IDAS - Social Anxiety	10.4(3.1)	8.9(4.4)	9.9(3.2)
ASI-3 - Total	21.6(12.8)	13.7(9.9)	24.5(12.2)

*Note*. Columns represent observed AB expressions (sub-groups) based on the >25 ms and <–25 ms BS cut-off. No data are presented for the no-bias sub-group because too few participants met this no-bias criteria to permit a reliable estimate and between-group comparison. *State- Trait Anxiety Inventory* (STAI) [35], *Overall Anxiety Severity and Impairment Scale* [40], *Inventory of Depression and Anxiety Symptoms* (IDAS) [41], and the *Anxiety Sensitivity Index-3* (*ASI-3*) [42].

### V. Gender and Attentional Bias Expressions: Descriptive Analysis

Recent work has shown gender differences in levels of AB [52]. Within the AB towards only sub-group there were 44.4% men (*N* = 16/36); within the Away only sub-group 31.8% were men (*N* = 7/22); and within the AB towards and away sub-group 22.2% were men (*N* = 8/36). These differences did not reach statistical significance (*χ^2^ = *4.04, *n.s*) though this analysis was likely underpowered.

## Discussion

The current study tested two central yet untested assumptions of the literature on attentional bias to threat. First, theories of attention to threat propose that high trait anxious individuals should demonstrate AB for multiple categories of threatening information [13], [16]. Moreover, extant study of AB has been conducted under the assumption that an anxious person expressing AB can exhibit *either* AB towards or AB away from threatening stimuli [2], [4], [11], [12]. The broad aim of the present investigation was to test these assumptions and better understand the nature of AB expression(s) to threat among high trait anxious adults. Such knowledge is important because an empirically-grounded model of the (endo)phenotypic expressions of AB to threat is key to advancing our measurement of the phenomenon(a), understanding its etiology, correlates, outcomes and means to therapeutically target AB and related anxiety psychopathology.

We observed an overall AB towards threat in high trait anxious individuals in line with previous studies [4]. However, upon closer inspection of the person-level expressions of AB in the sample, the dynamics of AB to threat emerge. Consistent with our MEAB hypothesis, we observed three expressions of AB to threat among trait anxious adults. Thirty-four percent demonstrated AB towards threatening stimuli; 20.8% demonstrated AB away from threatening stimuli; 34% demonstrated AB towards some categories of threat stimuli *and* AB away from other categories of threat stimuli; and 11.2% demonstrated no AB to threat. We then empirically tested and ruled out alternative explanations of the observed multiple expressions of AB phenomenon(a). We found that the observed multiple expressions of AB are not due to a specific criterion BS cut-off operationally defining AB, not specific to certain types of threat stimuli, nor an artifact of differences in within-subject variability in RT. Finally, we observed no significant differences in anxiety-related variables between participants who demonstrated each of the observed expressions of AB; accordingly, those expressing AB towards some *and* away from other categories of threat stimuli do not appear to exhibit lower risk for problems with anxiety relative to those demonstrating AB towards or away only.

These findings have a number of implications for the field’s conceptualization and future study of AB. First, observing multiple expressions of AB means that a uni-dimensional interval scale, distributional assumption (i.e., −100 ms to +100 ms) with an arbitrary value of 0 and single sample-level mean estimate of AB (i.e., mean sample AB = +10 ms) may not provide a valid representation of the nature nor levels of AB within a sample and intended population. Indeed, computing a sample-level mean of AB, by collapsing across sub-groups of individuals who demonstrate conflicting patterns of AB – some towards and others away from threat – forces a single interval scale with an arbitrary zero on a phenomenon(a) with a bi-dimensional ratio scale and a non-arbitrary or true zero. Thus, a **bi-dimensional ratio scale** is a more appropriate scale for a construct for which zero reflects the absolute lack of the phenomenon(a) (true zero = no AB), and for which high positive scores reflect high AB (bias towards) and high negative scores *also* reflect high AB (bias away). For example, it cannot be argued that +5 ms reflects *more AB than −*20 ms. Though in studies wherein a uni-dimensional interval scale with an arbitrary value of 0 and single sample-level mean are used to measure and study AB, this is what was done (cf., the proposed bi-dimensional ratio scaling with 0+/− as its point of bifurcation). Finally, because we observed that for each of the 5 studied categories of threat stimuli (i.e., angry faces, attacking dogs, attacking snakes, pointed weapons, violent scenes) some participants demonstrated AB towards and others away (Figures 2–4), the above described implication for uni-dimensional interval scaling vs. bi-dimensional ratio scaling of AB is potentially relevant to studies of AB, including studies that focus on only a single threat category (e.g., angry faces).

Second, observing that a significant proportion of trait anxious participants demonstrated AB towards *and* away from different threat stimuli may also have implications for the study of AB. The computation of AB which presumes that AB may be expressed as *either* towards only or away could result in potentially significant mis-estimation of AB. For example, participants exhibiting AB towards one category of threat (e.g., angry faces) and away from another category of threat (e.g., snakes) may, across categories of threat, be assigned an attenuated or near-zero AB score. Based on the present findings, we may infer that investigations which have operationalized threat by means of multiple types of stimuli (i.e., categories) may mis-classify ∼1/3 of the sample with respect to AB and consequently mis-estimate levels for AB for the sample as a whole. Accordingly, we propose that investigations of AB that include multiple threat stimuli compute one AB score per “category” of threat stimuli, each on a bi-dimensional ratio scale of AB (i.e., towards or away) with a true zero. Furthermore, investigations of AB that include only a single category of threat (e.g., angry faces) may need to consider the likelihood that observed AB towards only or away only may be a result of censored observations regarding other categories of threat which were not tested; indeed, were multiple threat categories tested, a substantive proportion of participants exhibiting either AB towards or away from a single category of threat stimuli may in fact demonstrate AB towards certain categories of threat stimuli and away from others. Accordingly, one possible implication of the present findings is that, in contrast to the majority of studies of AB, future investigations may benefit by inclusion of multiple categories of threat stimuli.

The present findings also highlight the potential significance of future research focused on the mechanisms underlying anxiety-related attentional bias. The observed pattern of variability in AB to threat in high anxious individuals is not in line with an oversensitive threat appraisal system non-discriminantly responding to multiple classes of threat. The current findings suggests that specific learning history (included in some models of AB, [13]) and specific contextual factors may exert an important influence on the expression of AB to threat. We propose that theories need to be refined to capture the dynamic nature of threat-related AB in anxiety.

Furthermore, a better understanding of the dynamics and specifically multiple expressions of AB may be important to understanding past and developing future research. Indeed, this more precise operationalization of AB may inform study of its etiology (e.g., genes, learning) [53]–[55], AB relations with variables of interest such as anxiety [4], neural substrate [56]–[58], as well as AB intervention outcomes (e.g., Attention Bias Modification Training (ABMT) [49], [50], [59], [60]; Attention Feedback Awareness and Control Training (A-FACT) [61]).

The present study has a number of limitations that qualify interpretation of the findings and may inform future research. First, a large probability-sample would provide more precise estimates of the prevalence of the observed multiple expressions of AB as well as a more representative sample of high-anxious adults, only estimated by the present university sample. Second, it is important that future work test the specificity/generalizability of these findings in non-anxious samples. Third, relatedly, the present findings beg the question regarding the MEAB hypotheses with respect to other forms of attentional bias and stimuli such as negative/positive emotion [62], [63], drug [29], or other aversive or appetitive stimuli. We theorize that the MEAB hypotheses will be similarly relevant for these other forms of AB. Fourth, the MEAB hypothesis was only tested by means of the modified dot probe paradigm and a single stimulus presentation duration (500 ms). We speculate that a longer duration (e.g., 750 ms) may increase the probability of observing both towards and away [30], [31], whereas a shorter duration (e.g., 200 ms) may reduce the probably of observing any AB away [32], [33], [64]. Furthermore, though used in the large majority of AB studies broadly and AB to threat more specifically, it is important to test the MEAB hypothesis across paradigms (e.g., spatial cueing, visual search) as well as measurement modalities (i.e., response time, eye-tracking). Finally, 16 trials per category of threat stimuli were tested. It may be useful for future study to replicate and extend the present observation of multiple expressions of AB with respect to a greater number of trials per category of stimuli. However, to the best of our knowledge, there are no published data indicating that 16 trials is psychometrically insufficient for estimation of AB per category of stimuli; nor reason to theorize that this number of trials is more likely to generate the observed MEAB effects relative to some (arbitrary) larger number of trials.

In summary, we found evidence consistent with the proposed Multiple Expressions of Attentional Bias Hypothesis (MEAB) – some trait anxious persons express AB towards threat and not away, others away from threat and not towards, yet others express AB towards *and* away concurrently to different categories of threat stimuli. To permit valid estimation of the observed multiple expressions of AB, we propose the field explore the implications of adopting a conceptual and operational bi-dimensional ratio scale of AB in contrast to the extant uni-dimensional interval scale. We hope that the proposed empirically-grounded model of the expressions of AB to threat will contribute to understanding the nature of AB, and perhaps ultimately, help advance our knowledge of the phenomenon(a), its etiology, correlates, outcomes and interventions.
